# Pharmacology education embedded within longitudinal community patient partnerships: an interprofessional approach to medication safety and clinical application

**DOI:** 10.3389/fphar.2026.1891970

**Published:** 2026-07-09

**Authors:** Olivia Henderson, Helen Hoye, Skylar Whittenbarger, Emma Lowe, Ariane Burt, Emma Norrod, Kelly Karpa

**Affiliations:** Department of Medical Education, Quillen College of Medicine, East Tennessee State University, Johnson city, TN, United States

**Keywords:** competency-based medical education, longitudinal learning, medication safety, patient-centered, pharmacology, undergraduate medical education

## Abstract

Pharmacology medical education is typically delivered in decontextualized contexts that emphasize *memorization* rather than *application* within patient care settings. Competency-based medical education frameworks increasingly emphasize observable clinical abilities related to prescribing, medication safety, and interprofessional collaboration, yet learners may have limited opportunities to practice these skills. In a longitudinal partnership between community volunteers (“patients”) and health profession students that involved monthly co-learning experiences centered around health and wellness topics, medication safety was identified as a highlight for both student and patient participants. During medication reviews and discussions with patients, potential medication-related problems were identified and classified. This perspectives paper describes a longitudinal, interprofessional, patient-centered model for contextualized pharmacology education. Embedding pharmacology learning within real-world patient interactions may provide early interprofessional learners with meaningful opportunities for application of pharmacologic principles, medication safety awareness, collaborative clinical reasoning, and may facilitate development of competencies aligned with competency-based medical education frameworks.

## Introduction

Pharmacology education remains a cornerstone of medical training but is often delivered in decontextualized formats that emphasize memorization and factual recall rather than patient-centered application. Although students may demonstrate competency in classroom-based assessments and standardized examinations, they may struggle to apply pharmacologic principles within real-world patient care settings (Johannsen et al., 2019). Importantly, even postgraduate resident physicians report deficits in pharmacology knowledge and prescribing preparedness, underscoring the need for more application-oriented educational approaches (Tobaiqy et al., 2025; Johannsen et al., 2019). The disconnect between classroom-based pharmacology learning and real-world clinical application may contribute to prescribing errors, inadequate medication counseling, and difficulty recognizing drug interactions and therapeutic alternatives (Maxwell and Walley, 2003).

Challenges with pharmacology education are particularly relevant within competency-based medical education (CBME). Organizations including the Association of American Medical Colleges (AAMC), Accreditation Council for Graduate Medical Education (ACGME), American Association of Colleges of Osteopathic Medicine (AACOM), and the Royal College of Physicians and Surgeons of Canada increasingly emphasize demonstration of observable clinical competencies within authentic patient care environments rather than isolated acquisition of factual knowledge (Frank et al., 2010; AAMC, 2026). Despite increasing emphasis on Entrustable Professional Activities (EPAs), patient safety, and interprofessional competencies, pharmacology education often remains centered on classroom-based knowledge acquisition rather than longitudinal application of medication-related decision-making within real patient interactions (AAMC, 2014). Consequently, learners may have limited opportunities to develop prescribing readiness, medication counseling skills, and systems-based approaches to medication safety before entering clinical training environments.

Experiential learning theory suggests that knowledge is retained more effectively when learners apply it in clinical contexts (Kolb, 1984). Longitudinal patient engagement offers a valuable opportunity for students to connect pharmacologic principles with real-world medication use, particularly among patients with complex medication regimens and polypharmacy. In parallel, interprofessional education (IPE) has been shown to strengthen collaborative competencies and team-based care skills. Integrating pharmacology education into longitudinal, interprofessional interactions centered around patients may provide an effective framework for developing clinical reasoning, communication, and collaborative practice skills. Importantly, such a model also promotes reciprocal learning - that benefits both students and patients - by supporting patient understanding of medication use and safety.

Situated learning theory suggests that placing students in clinical environments early in training promotes the development of critical thinking and clinical reasoning skills important for safe and effective pharmacotherapeutic decision-making (Firman et al., 2025). Similarly, the community of inquiry framework emphasizes that educational experiences incorporating social interaction, cognitive engagement, and teaching presence can move learners beyond rote memorization toward deeper understanding and application of knowledge (Collins and McLain, 2021). Prior work in pharmacology education has similarly suggested that case-based and problem-based learning approaches promote cognitive presence by requiring learners to consider comorbidities, therapeutic priorities, and patient-specific factors, thereby helping bridge the gap between foundational pharmacology knowledge and clinical application (Karpa and Vrana, 2013). Together, these frameworks support educational models that integrate pharmacology learning within collaborative, patient-centered clinical experiences.

By engaging students in longitudinal partnerships with patients, it may be possible to operationalize experiential, situated, and competency-based learning principles in the context of authentic medication-related encounters where students can participate in medication-focused discussions, recognize potential medication-related problems, and apply pharmacologic reasoning while simultaneously developing communication, teamwork, and medication safety skills. Such experiences may support development of competencies associated with EPA 4, including medication reconciliation, safe prescribing discussions, and justification of medication rationale, while also fostering collaborative practice competencies outlined by the Interprofessional Education Collaborative (AAMC, 2014; Interprofessional Education Collaborative, 2023). Prior skill-based medication safety curricula have demonstrated that active, applied pharmacology instruction can positively influence prescribing behaviors and medication safety practices (Karpa et al., 2015).

Building upon these educational frameworks, a longitudinal, interprofessional pharmacology learning experience was embedded within a patient–student partnership model. The initiative was intended to create opportunities for students to engage in medication-related discussions, communication, and patient education in real-world contexts. In this Perspectives paper, we highlight features of this model and discuss preliminary findings suggesting that students and patients place high value on the medication safety component of this experience, and the opportunity provided students with the chance to apply pharmacologic concepts and identify potential medication-related problems within patient-centered settings.

## Patient-student partnership model

The Patient-Student Partnership Pathway program at the Quillen College of Medicine is a volunteer, co-curricular experience established to provide health professions students with opportunities to engage longitudinally with community member volunteers (e.g., their “patient”), fostering continuity-based learning, patient-centered communication, understanding of social and environmental factors affecting health, and application of clinical knowledge within community contexts.

Health professions students were organized into pairs and assigned a community member “patient” whom they visited monthly in the patient’s home over an 8-month period. Each month centered on a specific health and wellness theme. Students prepared for home visits by completing microteaching modules that were pre-recorded and housed within our institution’s learning management system. Students subsequently applied concepts from microteachings when visiting their patient. After home visits in which students practiced obtaining health and wellness histories, student teams encouraged their “patient” to develop a health/wellness SMART (specific, measurable, achievable, relevant, and time-bound) goal; students served as accountability partners for their patient by reviewing progress throughout the program. Additional home visits focused on topics relevant to older adults (aged 65 and older) including nutrition, home safety, medication management, and end-of-life planning themes.

## Medication-focused home visits

Students were accompanied to medication-focused visits by a faculty member who is a professor (PhD) and pharmacist (RPh). These visits were standardized in overall structure while allowing flexibility to address each patient’s unique medication regimen and clinical circumstances.

Prior to the medication review visit, student teams collected information regarding each patient’s current medications, including medication names, dosages, indications, and relevant health history. This information was provided to the pharmacist in advance of the encounter. Using these materials, the pharmacist prepared individualized educational handouts for each patient that included medication-specific information and visual illustrations of drug mechanisms of action.

Each medication review followed a consistent format across all patient encounters. The pharmacist first engaged patients in discussion regarding their medications, asking questions such as *why* they believed a medication had been prescribed. The pharmacist then engaged the student team in guided discussion designed to connect foundational pharmacology concepts to the patient’s real-world clinical situation. Students were asked to explain medication mechanisms of action, therapeutic rationale, adverse effects, drug interactions, and disease-specific treatment strategies. For example, students might be asked to explain to patients why multiple medications were being used to treat hypertension or to predict adverse effects based on a medication’s mechanism of action. Students were also encouraged to provide patient education and clarification when appropriate.

Although the educational discussion was individualized to each patient’s medications and health conditions, the pharmacist’s role and level of involvement was consistent across all medication review encounters. The pharmacist facilitated each session, guided questioning, promoted discussion between patients and students, and helped identify opportunities for medication-related education and clinical reasoning.

**FIGURE 1:**
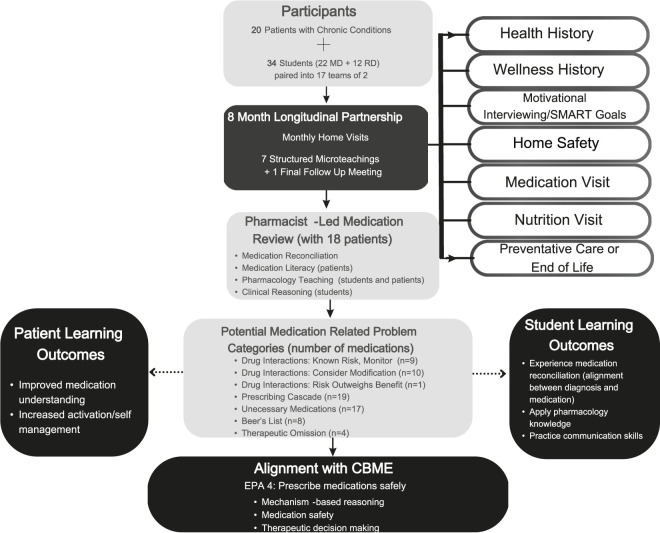
Overview of the pharmacist-led medication review component of the longitudinal Patient–Student Partnership program. Students and patients participated in an 8-month series of home visits, including a structured pharmacist-led medication review. Medication-related problems identified during the review served as learning opportunities that enhanced patient medication understanding and supported student development of pharmacology knowledge, clinical reasoning, communication skills, and competency-based prescribing skills. The activity aligned with CBME outcomes related to medication safety and therapeutic decision-making. MD, medical student; RD, dietitian student; CBME, competency based medical education; EPA, entrustable professional activity.

Medication-related problems were identified collaboratively during the review process through discussion among the patient, student team, and pharmacist. Following each encounter, student teams documented potential medication-related issues that had been identified or discussed. Members of the student research team subsequently reviewed these documented issues and categorized them according to the categories reported in Figure 1. Categorization was performed using consensus review among the students and the pharmacist. Because the categories were applied retrospectively for descriptive reporting purposes, no formal calibration exercise was conducted prior to categorization.

Neither the students nor the pharmacist recommended medication changes to patients; all healthcare decisions remained between the patients and their healthcare providers. When appropriate, the pharmacist assisted community volunteers in identifying questions that could be discussed with healthcare providers during future appointments.

For programmatic quality improvement purposes, students and patients were invited to complete questionnaires prior to- and at the conclusion of-the program to ascertain which components of the overall patient-student partnership were most impactful.

During the 2025–2026 academic year, 34 students from medical (first year; n = 22) and dietitian (first year graduate learners; n = 12) professions were paired into 17 teams and matched with 20 community-dwelling older adults, including three husband/wife pairs. Formal medication reviews were conducted with 18 patients (two spouses did not submit a medication list for review) (Figure 1).

## Pharmacology principles applied to patients

Cumulatively, during medication visits with patients, 68 potential medication-related problems (average of 3.8 per patient; minimum = 0; maximum = 11) were discussed between the pharmacist and student teams. Students categorized the potential medication-related problems into five categories: drug interactions (3 distinct levels), prescribing cascade events, potentially unnecessary medications due to lack of therapeutic rationale, Beer’s list medications that are potentially inappropriate in elderly individuals, and therapeutic omissions.

Adverse events caused by medications were a prevalent issue discussed; six of the patients experienced a known side effect from prescribed medications. Often, instead of having a medication switched when an adverse effect occurred, another drug had been added to treat the new adverse event symptom. Another common medication-related problem identified in this population involved medications that seemed to be unnecessary and lack therapeutic rationale. For example, over 20% of patients (4 of 18) affected by potential medication-related problems were prescribed duplicate therapies to treat gastroesophageal reflux even though the combination was unlikely to provide additional benefits but could increase cost and unwanted effects. Additionally, contraindications and therapeutic omissions were also frequently encountered and discussed. In all, students identified at least one potential medication-related problem in 15 of 18 patients (83%), highlighting the value of interprofessional pharmacology education in promoting patient-centered medication safety while simultaneously providing learners with authentic opportunities to apply pharmacology knowledge, collaborate across professions, and develop clinical reasoning and communication skills within the context of real patients rather than isolated classroom-based learning.

## Student and patient perceptions of the pharmacology component of the program

Sixteen students responded to a question at the end of the Patient-Student partnership program that requested feedback about the most important thing learned from all the modules. Eight (50%) of the respondents (5 medical students; three dietitian students) cited aspects related to pharmacology. Similarly, patients were asked which of the monthly themes had the most positive impact on them. Eleven individuals responded to this question, with five (45%) referencing the medication safety session as most valuable, including two individuals who had participated in previous iterations of the program (before data collection commenced).

Of the 12 patient respondents for whom matched pre/post data were available for a question modified from the Patient Activation Measure instrument (PAM) (“I know what each of my prescribed medications do”), none indicated a decreased level of agreement over the course of the program on the four-point Likert-based tool (Hibbard et al., 2007). Several participants demonstrated increased agreement, including one respondent who changed from disagreeing to strongly agreeing with the statement. No matched respondents demonstrated decreased agreement. At the conclusion of the program, all respondents either agreed or strongly agreed that they understood what their medications did for them. Improvements in agreement were observed in both first-time and repeat participants, although the largest change (disagree shifting to strongly agree) occurred in a first-time participant (Table 1).

**TABLE 1:** Transition table of patient responses to PAM medication item from pre-to post-program participation. PAM, patient activation measure.

Pre-program response	Post-program response	n	1st-time participants	Prior participants
Disagree	Strongly agree	1	1	0
Agree	Agree	2	1	1
Agree	Strongly agree	2	1	1
Strongly agree	Strongly agree	7	4	3

These findings suggest that pharmacology—particularly in the context of medication safety reviews—emerged as a highly meaningful and valued component of the longitudinal experience for both learners and patients. Notably, both medical and dietitian students independently identified pharmacology-related learning as one of the most important aspects of the experience. This cross-disciplinary engagement suggests that the module reinforced pharmacologic principles in ways that were meaningful across professional roles rather than being perceived as isolated or discipline-specific. Instead, medication-related discussions functioned as a shared clinical and collaborative learning space in which learners applied pharmacologic reasoning within authentic patient contexts.

Patient responses further reinforced the perceived value of medication-related content within the program. Nearly half of respondents identified the medication safety session as having the greatest positive impact, suggesting that medication-focused discussions resonated with the community volunteers and may address unmet needs related to medication understanding and engagement. Notably, two of these responses came from individuals who had participated in earlier iterations of the program prior to formal data collection, suggesting that the perceived value of this content may extend across multiple cohorts of community members (not only new program participants). These findings are particularly relevant given that medication-related problems remain a major health concern among older adults, especially those with complex medication regimens and polypharmacy. Prior community-based medication education initiatives, such as the Community Medication Education, Data, and Safety (C-MEDS) program, have similarly demonstrated that personalized medication safety education can improve medication self-efficacy and adherence among community-dwelling older adults (Meyer et al., 2021).

Together, these findings align with interprofessional education literature demonstrating that medication safety–focused IPE activities are well received across health professions and can strengthen collaborative competencies related to communication, teamwork, and patient-centered care (Grimes et al., 2023). Similarly, interprofessional medication management experiences involving medical and other health professions students have been associated with improvements in attitudes toward collaboration and confidence in medication-related tasks (Kostas et al., 2018). The longitudinal nature of the program may also have contributed to these outcomes, as continuity-based educational experiences have been shown to enhance learner confidence, patient-centeredness, and integration of knowledge and skills across clinical domains (Hirsh et al., 2012).

These observations are also consistent with CBME frameworks that emphasize observable application of knowledge within authentic clinical environments rather than isolated demonstration of factual recall (Frank et al., 2010). Within pharmacology education, CBME requires learners not only to understand pharmacologic mechanisms, but also to apply medication knowledge to patient-centered decision-making, communication, and medication safety practices (Frank et al., 2010; Englander et al., 2016). The longitudinal patient-student partnership model described herein provides opportunities for learners to identify medication-related problems, recognize prescribing cascades and potentially inappropriate therapies, and discuss medications within interprofessional patient-centered encounters. Such experiences may help support development of competencies associated with EPA 4, including discussing medications and prescriptions with patients and healthcare team members (Association of American Medical Colleges [AAMC], 2014).

Importantly, this model positions pharmacology learning within the context of real patients, complex medication regimens, and authentic medication-related concerns rather than isolated classroom or simulation-based instruction. In contrast to traditional pharmacology education, competency-based and contextualized approaches emphasize integration of pharmacology knowledge with clinical reasoning, patient communication, and systems-based medication management (Firman et al., 2025; Collins and McLain, 2021). Prior literature has suggested that traditional classroom-based pharmacology is less effective in fostering critical thinking and problem-solving skills (Blouin et al., 2008). Learning within authentic clinical contexts may also enhance retention and transfer of knowledge (Blouin et al., 2008). The medication-related problems identified and discussed within this program suggest that longitudinal, patient-centered pharmacology experiences may create meaningful opportunities for early development of medication safety and prescribing competencies while reinforcing foundational pharmacologic principles in real-world environments (Karpa et al., 2015).

Additionally, the Patient–Student Partnership model incorporates elements of co-production in which students and educators collaboratively shape the learning experience through ongoing feedback, discussions, and shared teaching interactions with community members. Co-production in education has been associated with improved student–faculty relationships, enhanced institutional culture, and positive effects on students’ professional and personal development (Gheihman et al., 2021). It has also been shown to increase learner engagement (Gheihman et al., 2021), an important factor given the established relationship between student engagement, academic performance, and development of lifelong learning behaviors (Oyler et al., 2016).

This initiative involved a relatively small number of participants at a single institution and included primarily descriptive and perception-based outcomes. Because of the voluntary nature of participation, both students and community members may have been more motivated and engaged than broader populations. Additionally, we did not have a control group of students or patients, nor did we utilize an objective assessment of pharmacology knowledge, retention, or prescribing competence. The potential medication-related problems noted through student discussions were educational observations rather than clinical interventions. As such, these findings should be interpreted as preliminary observations supporting the feasibility and perceived value of contextualized pharmacology learning rather than definitive evidence of educational or clinical effectiveness.

Despite these limitations, the findings highlight important implications for pharmacology and health professions education. Pharmacology is frequently taught as an isolated foundational science discipline, yet medication-related decision-making occurs within complex interpersonal, clinical, and system-based contexts. Embedding pharmacology learning within longitudinal, interprofessional patient partnerships may provide learners with early opportunities to apply pharmacological principles to actual medication-related concerns while simultaneously reinforcing the communication, teamwork, and medication safety competencies that are emphasized in CBME frameworks. Additionally, the positive responses from community participants suggest that medication-focused educational interactions may represent a mutually beneficial learning strategy that supports both learner development and patient engagement.

Future work may explore more rigorous assessment of longitudinal, contextualized pharmacology education models, including objective measures of medication safety skills and prescribing readiness (Maxwell et al., 2015). Additional studies involving larger and more diverse learning populations across multiple institutions would help determine generalizability and scalability of this approach. Longitudinal follow-up into clinical clerkship or residency training may also help clarify whether early patient-centered pharmacology experiences influence subsequent prescribing readiness, medication safety practices, and interprofessional clinical engagement.

## Data Availability

The raw data supporting the conclusions of this article will be made available by the authors, without undue reservation.
